# Interpretable machine learning-based prediction of 28-day mortality in ICU patients with sepsis: a multicenter retrospective study

**DOI:** 10.3389/fcimb.2024.1500326

**Published:** 2025-01-08

**Authors:** Li Shen, Jiaqiang Wu, Jianger Lan, Chao Chen, Yi Wang, Zhiping Li

**Affiliations:** ^1^ Department of Clinical Pharmacy, Children’s Hospital of Fudan University, National Children’s Medical Center, Shanghai, China; ^2^ Department of Pharmacy, Suzhou Hospital, Affiliated Hospital of Medical School, Nanjing University, Suzhou, Jiangsu, China; ^3^ School of Life Sciences and Biopharmaceutical Science, Shenyang Pharmaceutical University, Shenyang, China; ^4^ Department of Neonatology, Children’s Hospital of Fudan University, National Children’s Medical Center, Shanghai, China; ^5^ Department of Neurology, Children’s Hospital of Fudan University, National Children’s Medical Center, Shanghai, China

**Keywords:** machine learning, sepsis, 28-day mortality, multicenter retrospective study, XGBoost

## Abstract

**Background:**

Sepsis is a major cause of mortality in intensive care units (ICUs) and continues to pose a significant global health challenge, with sepsis-related deaths contributing substantially to the overall burden on healthcare systems worldwide. The primary objective was to construct and evaluate a machine learning (ML) model for forecasting 28-day all-cause mortality among ICU sepsis patients.

**Methods:**

Data for the study was sourced from the eICU Collaborative Research Database (eICU-CRD) (version 2.0). The main outcome was 28-day all-cause mortality. Predictor selection for the final model was conducted using the least absolute shrinkage and selection operator (LASSO) regression analysis and the Boruta feature selection algorithm. Five machine learning algorithms including logistic regression (LR), decision tree (DT), extreme gradient boosting (XGBoost), support vector machine (SVM), and light gradient boosting machine (lightGBM) were employed to construct models using 10-fold cross-validation. Model performance was evaluated using AUC, accuracy, sensitivity, specificity, recall, and F1 score. Additionally, we performed an interpretability analysis on the model that showed the most stable performance.

**Results:**

The final study cohort comprised 4564 patients, among whom 568 (12.4%) died within 28 days of ICU admission. The XGBoost algorithm demonstrated the most reliable performance, achieving an AUC of 0.821, balancing sensitivity (0.703) and specificity (0.798). The top three risk predictors of mortality included APACHE score, serum lactate levels, and AST.

**Conclusion:**

ML models reliably predicted 28-day mortality in critically ill sepsis patients. Of the models evaluated, the XGBoost algorithm exhibited the most stable performance in identifying patients at elevated mortality risk. Model interpretability analysis identified crucial predictors, potentially informing clinical decisions for sepsis patients in the ICU.

## 1 Introduction

Sepsis, a complex and life-threatening condition, arises from the host’s dysregulated response to infection, leading to organ dysfunction and potential mortality (Singer et al., 2016). Despite recent diagnostic and therapeutic advancements, sepsis continues to exhibit a high incidence and mortality rate. Annually, there are approximately 31 million cases of sepsis worldwide, with 5.5 million deaths (Rudd et al., 2020). In the United States, sepsis accounts for one of the top causes of in-hospital death, with around 750,000 cases per year and a mortality rate of up to 30% (Rhee et al., 2017). A study conducted at multiple centers in China found that 33.5% of sepsis patients in the ICU experienced a mortality rate within 28 days (Zhou et al., 2014). While scoring systems like APACHE II and SOFA are commonly employed to predict outcomes in critically ill patients, including those with sepsis, they were not specifically designed for sepsis populations (Singer et al., 2016). Consequently, their predictive accuracy for sepsis-related mortality has been found to be suboptimal.

Several studies indicated that age, underlying diseases, infection site, and organ dysfunction severity were key risk factors influencing sepsis prognosis (Liu et al., 2021; Cui et al., 2024). Among these, age ≥65 years, comorbid chronic diseases, unclear or multiple infection foci, APACHE II score ≥25, and SOFA score ≥10 are closely related to poor prognosis in sepsis patients (Yang et al., 2023; Cui et al., 2024). Additionally, recent studies have found that serum lactate levels, coagulation abnormalities, and immune dysfunction are also important factors affecting sepsis (S, 2022; Li et al., 2023; Kim et al., 2024). Septic shock was a significant mortality risk factor (Hotchkiss et al., 2016), and elevated inflammatory mediators like IL-6 and procalcitonin were linked to poor outcomes (Liu et al., 2021). Furthermore, specific gut microbiome signatures have been linked to increased mortality risk (Yang et al., 2024). In summary, sepsis remains a prevalent and deadly condition with a multifactorial risk profile. Identifying risk factors and developing predictive models are essential for enhancing sepsis patient outcomes.

In the last few years, machine learning (ML) algorithms have proven to be highly effective in predicting mortality risk for ICU patients suffering from sepsis. A recent study has developed and validated a stacking ensemble ML model that effectively predicts the in-hospital mortality risk for patients suffering from sepsis-induced coagulopathy. Based on data from the MIMIC-IV database, the model identified anion gap and age as the most crucial predictive features (Liu et al., 2024). Zhou S. et al (Zhou et al., 2024) constructed an XGBoost model based on 17 features that demonstrated good generalizability across multiple external datasets. Another study (Wang et al., 2022) found that the LightGBM model outperformed other ML algorithms in predicting 30-day mortality for sepsis patients, achieving an AUC of 0.90. These studies indicate that machine learning methods can integrate multidimensional clinical information to provide more accurate individualized predictions. However, existing research still has some limitations. Firstly, most models lack interpretability, making it difficult for clinicians to fully understand and trust them (Gao et al., 2024). Secondly, many studies deal with missing values to varying degrees, using algorithms for imputation or processing (Li et al., 2024). Although these algorithms are scientifically based, it is unavoidable that the imputed data are virtual.

**Figure 1:**
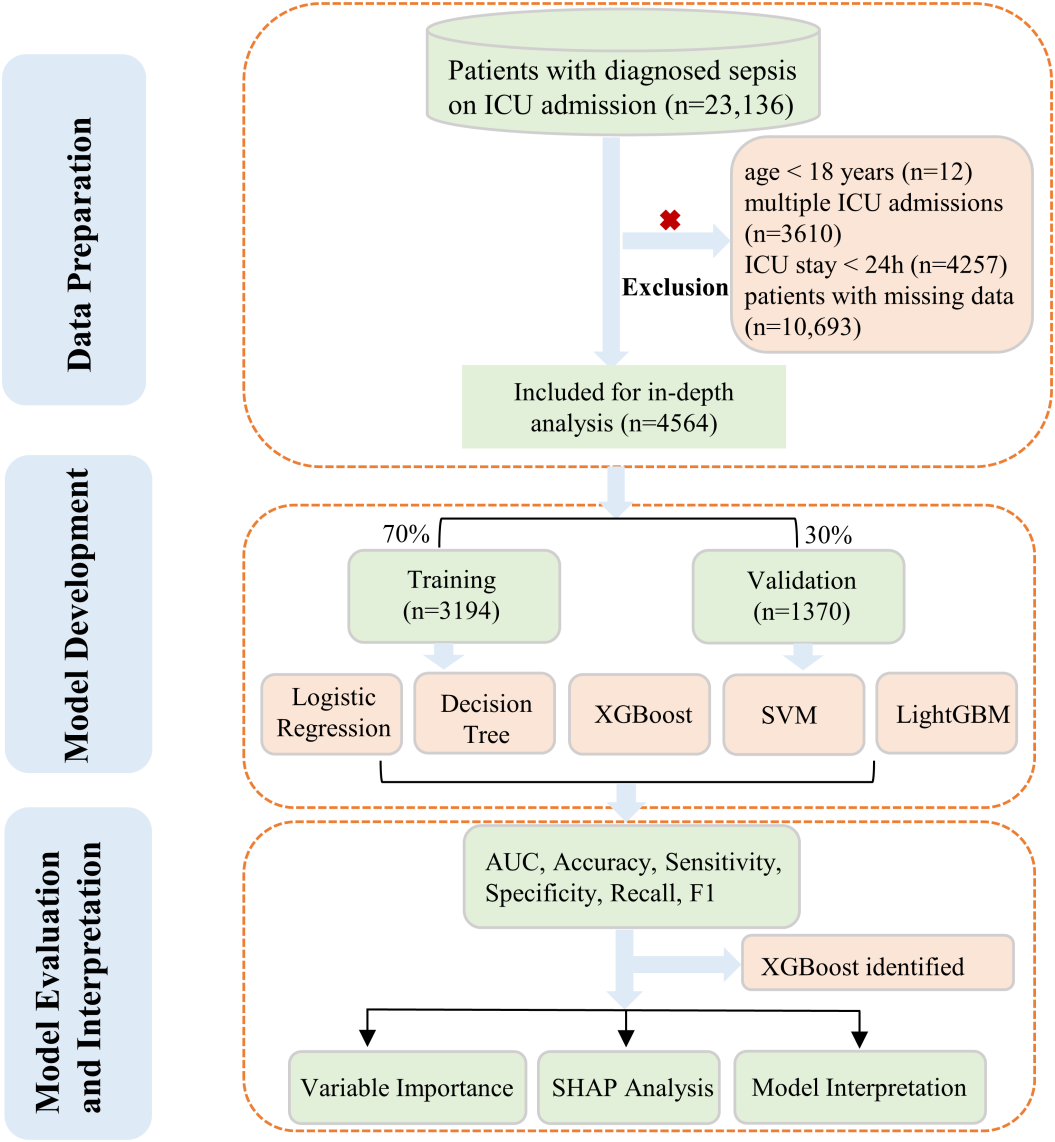
The whole study workflow.

Our study focused on the development and evaluation of five distinct ML algorithms. These models were designed to predict the likelihood of death from any cause within 28 days for sepsis patients admitted to ICU. To achieve this, we utilized the comprehensive eICU database for in-depth analysis. We used completely authentic clinical variables without imputation to ensure data reliability and better represent real-world scenarios. Additionally, we conducted interpretability analyses on the model with the most stable performance to enhance clinical applicability. The complete workflow was presented in **Figure 1**.

## 2 Methods

### 2.1 Data source

All the data was derived from the eICU Collaborative Research Database (eICU-CRD). The eICU-CRD (https://eicu-crd.mit.edu), a large multi-center critical care database made available by Philips Healthcare in partnership with the MIT Laboratory for Computational Physiology, is a de-identified, freely accessible dataset containing information from 200,859 ICU patient admissions across 208 U.S. hospitals, compiled to facilitate research in critical care (Pollard et al., 2018). Because of its retrospective nature, lack of direct patient intervention, and adherence to safe harbor standards for data de-identification, this research was deemed exempt from the Massachusetts Institute of Technology’s institutional review board approval. The de-identification process was certified by Privacert (Cambridge, MA) as compliant with the Health Insurance Portability and Accountability Act (Certification no. 1031219-2), ensuring minimal risk of subject re-identification. The author L.S. has completed a certification course (Record ID: 54499751) sanctioned by the PhysioNet review committee, has database access, and was responsible for extracting data following the data usage agreement.

### 2.2 Participants

Inclusion criteria for this study encompassed sepsis patients aged 18 years and above. For individuals with multiple ICU stays, only the initial admission was considered in our analysis. Sepsis was defined according to criteria established by the Third International Consensus Definitions for Sepsis and Septic Shock (Sepsis-3) (Singer et al., 2016). In our study, patients with an ICU stay duration of less than 24 hours were not included. Furthermore, we excluded cases lacking documented ICU outcomes to maintain result integrity. Lastly, subjects with incomplete or missing data entries were omitted from the analysis to prevent potential bias and ensure robust findings. The outcome was all-cause ICU mortality within 28 days after being admitted.

### 2.3 Feature extraction

Baseline characteristics were extracted over the initial 24-hour period after ICU admission. Data on demographics, vital signs, severity score of illness, laboratory tests, and comorbidities or not were analyzed in this study. Demographics contain age, gender, admission weight, body mass index (BMI), report year, and ethnicity. Vital signs include temperature, respiratory rate, heart rate, and mean arterial pressure (MAP). Severity score of illness including Glasgow Coma Scale (GCS) score, Sequential Organ Failure Assessment (SOFA) score, Acute Physiology III Score, and Acute Physiology and Chronic Health Evaluation (APACHE) IV Score. Laboratory test data including blood urea nitrogen, alkaline phosphatase (ALP), glucose, blood sodium, serum creatinine, aspartate aminotransferase (AST), alanine aminotransferase (ALT), total bilirubin, total protein, albumin, lactate, platelets, red blood cell, mean corpuscular hemoglobin concentration (MCHC), hemoglobin, red cell distribution width and white blood cell count (WBC). Comorbidities include chronic obstructive pulmonary disease (COPD), congestive heart failure, acute myocardial infarction (AMI), diabetes, pneumonia and rhythm disturbance.

### 2.4 Statistical analysis

The study population was randomly split into a 70% training set and a 30% validation set. **Supplementary Table S1** in the **Supplementary Material** presented the detailed information of the two sets.

We employed a two-step feature selection process to identify the most relevant variables for our predictive model. Initially, we applied the least absolute shrinkage and selection operator (LASSO) regression, a method that performs variable selection and coefficient shrinkage through regularization (Alhamzawi and Ali, 2018). LASSO regression utilized 10-fold cross-validation to determine the optimal lambda value that minimized the mean cross-validated error (McNeish, 2015). Lambda (λ) is a tuning parameter that controls model complexity and the stringency of feature selection, where smaller values retain more features in the model. Subsequently, we implemented Boruta feature selection (Ejiyi et al., 2024), an algorithm based on the random forest that identifies all relevant variables by comparing the importance of original features with randomly generated “shadow features”. The Boruta algorithm was executed with 1000 iterations and a p-value threshold of 0.05. To ensure a robust and parsimonious model, we selected the intersection of features identified by both LASSO regression and the Boruta algorithm as our final set of predictor variables. Five ML algorithms were employed to construct models: logistic regression (LR), decision tree (DT), extreme gradient boosting (XGBoost), support vector machine (SVM), and light gradient boosting machine (ligthGBM). Model development utilized 10-fold cross-validation to enhance reliability and generalizability. Performance evaluation of the models encompassed multiple metrics: the area under the receiver operating characteristic curve (AUC), accuracy, sensitivity, specificity, recall, and F1 score. For all of these performance indicators, the values range from 0 to 1, with higher scores indicating better model performance. Following the comprehensive evaluation of model performance, we selected the model demonstrating the highest stability across all performance metrics as our final predictive model. To enhance the interpretability of this model, we conducted Shapley Additive Explanations (SHAP) analysis, a game theory-based approach that attributes feature importance to individual predictions (Guo et al., 2023).

To guarantee the veracity of the data included in the study, any variables with missing data were excluded. For continuous variables, we reported either the mean accompanied by its standard deviation (SD) or the median with its corresponding interquartile range (IQR), depending on the distribution pattern. In contrast, categorical data were displayed as counts and their respective percentages. To assess differences between groups, we employed distinct statistical methods. Continuous variables underwent analysis using one-way ANOVA, while categorical data were examined through chi-square testing. Our statistical approach maintained a two-tailed perspective, with significance established at P < 0.05.

All statistical analyses and data visualizations were performed using R software package (version 4.2.1). For feature selection, we employed LASSO regression using the “glmnet” package and Boruta algorithm using the “Boruta” package. Model development was conducted using multiple packages: LR was implemented using “glm”, DT using “rpart”, XGBoost using “xgboost”, SVM using “e1071”, and LightGBM using “lightgbm”. SHAP values were calculated and visualized using the “shapviz” package.

## 3 Results

### 3.1 Baseline characteristics

A total of 4,564 eligible patients were eventually enrolled, with 3,194 individuals in the training set and 1,370 in the validation set. The survivor and non-survivor groups were categorized according to whether or not an all-cause death occurred within 28 days of admission to the ICU. **Table 1** presented the baseline characteristics of survivors (n=996) and non-survivors (n=568). Compared to survivors, non-survivors were older (67.56 ± 13.52 vs 64.91 ± 15.94 years, P<0.001), had lower body weight (80.19 ± 27.05 vs 83.37 ± 27.67 kg, P=0.01), and lower BMI (28.12 ± 8.87 vs 29.18 ± 9.03, P=0.008). Non-survivors exhibited more abnormal vital signs, including lower temperature, higher respiratory rate, and faster heart rate (all P<0.001). Severity scores were significantly higher in the non-survivor group, such as SOFA (7.00 vs 5.00, P<0.001) and APACHE score (97.00 vs 72.00, P<0.001). Laboratory tests revealed more severe renal and hepatic dysfunction in non-survivors, with notably higher lactate levels (P<0.001). Regarding comorbidities, non-survivors had higher rates of congestive heart failure (P=0.024) and pneumonia (P=0.004), but lower rates of diabetes (P=0.012). These findings highlight several key clinical and laboratory parameters associated with mortality in our study population.

**Table 1:** Demographics and baseline characteristics.

Parameters	Survivors (n = 3996)	Non-survivors (n = 568)	P-value
Demographics			
Age, years	64.91 ± 15.94	67.56 ± 13.52	<0.001
Gender			0.521
Male	1950 (48.80%)	269 (47.36%)	
Female	2046 (51.20%)	299 (52.64%)	
Admission weight, kg	83.37 ± 27.67	80.19 ± 27.05	0.01
BMI	29.18 ± 9.03	28.12 ± 8.87	0.008
Report year			0.464
2014	1661 (41.57%)	237 (41.73%)	
2015	2335 (58.43%)	331 (58.27%)	
Ethnicity			0.296
Caucasian	3156 (78.98%)	459 (80.81%)	
African American	367 (9.18%)	51 (8.98%)	
Hispanic	234 (5.86%)	31 (5.46%)	
Asian	123 (3.08%)	8 (1.41%)	
Native American	39 (0.98%)	8 (1.41%)	
Other/Unknown	77 (1.93%)	11 (1.94%)	
Length of Stay in ICU, days	3.08 (1.93-5.73)	3.09 (1.66-6.85)	0.739
Vital signs			
Temperature, °C	36.50 (36.10-36.90)	36.30 (35.70-36.70)	<0.001
Respiratory rate, bpm	32.00 (22.00-39.00)	35.00 (29.00-42.00)	<0.001
Heart rate,/min	116.00 (100.00-131.00)	123.50 (106.75-139.00)	<0.001
MAP, mmHg	56.00 (47.00-113.00)	50.00 (43.00-76.25)	0.370
Severity of illness			
GCS score	14.00 (10.00-15.00)	12.00 (7.00-15.00)	<0.001
SOFA score	5.00 (3.00-7.00)	7.00 (5.00-10.00)	<0.001
Acute Physiology Score III	57.50 (44.00-74.00)	81.00 (65.00-107.00)	<0.001
APACHE IV Score	72.00 (57.00-89.00)	97.00 (81.00-122.00)	<0.001
Laboratory data on day 1			
Blood urea nitrogen, mg/dL	28.00 (18.00-45.00)	38.00 (25.00-57.00)	<0.001
ALP, U/L	87.00 (63.00-127.25)	101.50 (72.00-162.00)	<0.001
Glucose, mg/dL	128.00 (102.00-172.25)	122.50 (95.75-167.25)	0.085
Blood sodium, mmol/L	138.00 (135.00-141.00)	138.00 (134.00-142.00)	0.950
Serum creatinine, mg/dL	1.40 (0.90-2.40)	1.91 (1.20-2.91)	<0.001
AST, U/L	37.00 (21.00-76.00)	67.00 (33.00-182.00)	<0.001
ALT, U/L	27.00 (17.00-55.00)	35.50 (20.00-91.25)	<0.001
Total bilirubin, mg/dL	0.70 (0.40-1.20)	1.00 (0.60-2.40)	<0.001
Total protein, g/dL	5.70 (5.20-6.30)	5.40 (4.60-6.00)	<0.001
Albumin, g/dL	2.50 (2.10-2.90)	2.20 (1.80-2.70)	<0.001
Lactate, mmol/L	1.80 (1.10-2.80)	3.10 (1.80-5.50)	<0.001
Platelets, ×10^9^/L	178.50 (121.00-251.00)	155.00 (78.00-234.00)	<0.001
Red blood cell, M/mcl	3.51 (3.02-4.01)	3.39 (2.91-3.88)	0.014
MCHC, g/dL	32.80 (31.80-33.70)	32.70 (31.60-33.70)	0.202
Hemoglobin, g/dL	10.40 (8.90-11.90)	10.10 (8.60-11.70)	0.263
Red cell distribution width, %	15.40 (14.20-17.12)	16.55 (14.97-18.52)	<0.001
White blood cell count, ×10^9^/L	13.90 (9.13-20.20)	14.55 (8.54-22.66)	0.004
Comorbidities			
COPD			0.889
No	3707 (92.77%)	526 (92.61%)	
Yes	289 (7.23%)	42 (7.39%)	
Congestive heart failure			0.024
No	3703 (92.67%)	511 (89.96%)	
Yes	293 (7.33%)	57 (10.04%)	
AMI			0.279
No	3850 (96.35%)	542 (95.42%)	
Yes	3850 (96.35%)	542 (95.42%)	
Diabetes			0.012
No	3433 (85.91%)	510 (89.79%)	
Yes	563 (14.09%)	58 (10.21%)	
Pneumonia			0.004
No	2744 (68.67%)	356 (62.68%)	
Yes	1252 (31.33%)	212 (37.32%)	
Rhythm disturbance			<0.001
No	3314 (82.93%)	438 (77.11%)	
Yes	682 (17.07%)	130 (22.89%)	

### 3.2 Feature selection

**Figure 2:**
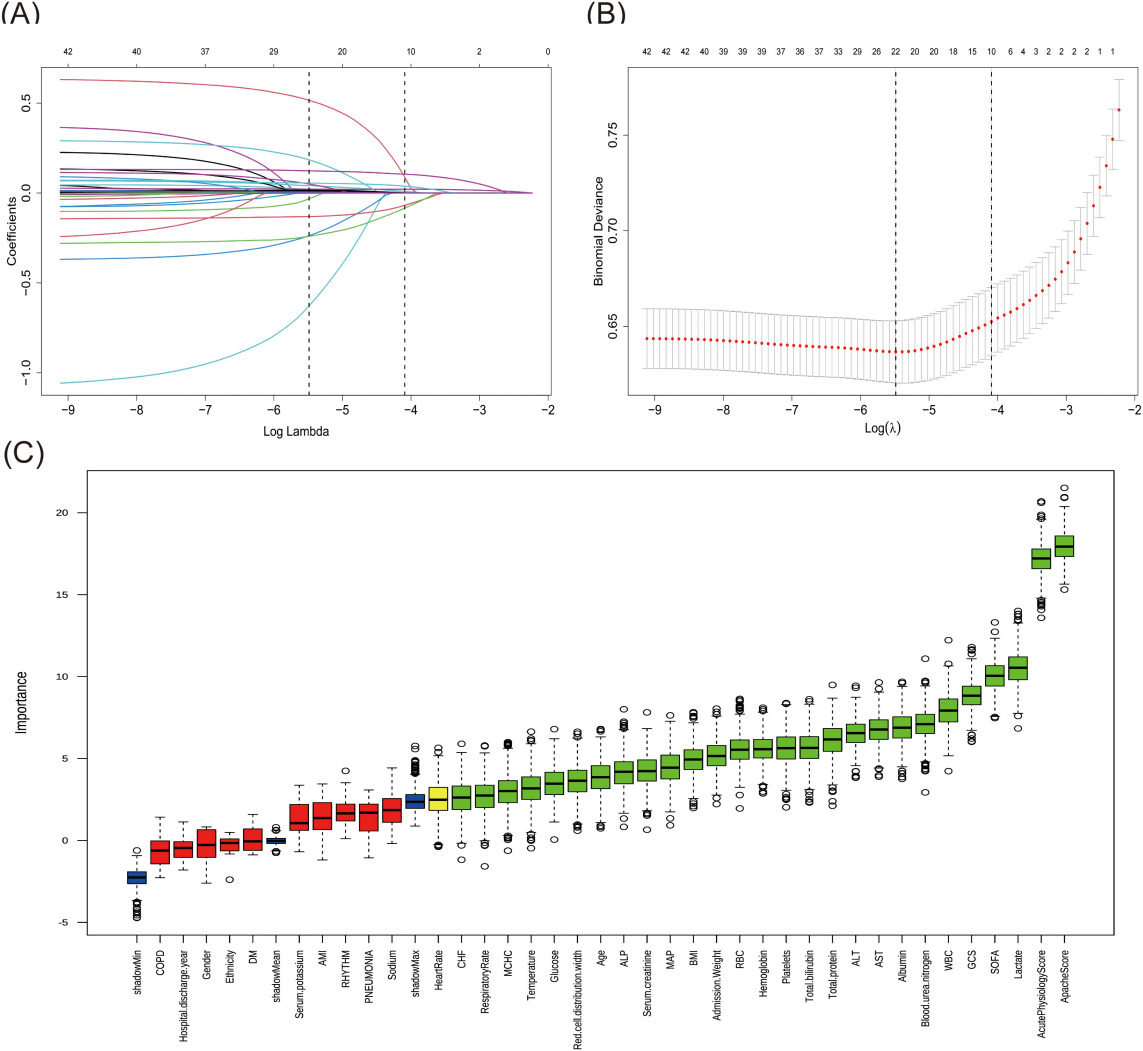
Features selection by LASSO regression and Boruta. **(A)** The variation characteristics of the LASSO coefficient. Selection of the optimal parameter Lambda (λ) in LASSO involved plotting log (λ) on the X-axis and regression coefficients on the Y-axis. The different colored lines represented the different variables. **(B)** Optimization parameters (λ) of the LASSO model were selected by 10-fold cross-validation. The left dashed line represents λmin (minimum cross-validated error), while the right dashed line indicates λ1se (the largest λ within one standard error of λmin). **(C)** Feature identification via Boruta algorithm. The X-axis represented all features, and the Y-axis was the Z-value of each feature. The green boxes represented the initial 26 significant variables, while the yellow ones denoted tentative, and the red ones indicated unimportant.

First, the LASSO regression analysis was performed with a sequence of lambda values (λ), where log(λ) ranged from -9 to -2. The Through 10-fold cross-validation, the optimal lambda value (lambda.min = 0.0041) was determined based on the minimum binomial deviance. As shown in **Figures 2A, B**, the upper x-axis numbers indicate the count of non-zero coefficient features retained at each lambda value, and two vertical dotted lines represent lambda.min (0.0041, left) and lambda.1se (0.0167, the largest lambda value within one standard error of the minimum), respectively. Using the optimal lambda.min, LASSO regression identified 22 significant variables with non-zero coefficients.The Boruta algorithm, used for characteristics screening, revealed after 1000 iterations a total of 28 variables, represented by green and yellow boxes in **Figure 2C**, which were found to be in front of shadowMax and were initially selected. By intersecting the variables derived from the two algorithms, a total of 17 variables were ultimately utilized in the construction of the ML model. These significant variables included age, admission weight, respiratory rate, GCS score, SOFA score, APACHE score, CHF, blood urea nitrogen, ALP, glucose, AST, total bilirubin, total protein, albumin, lactate, red cell distribution width and WBC.

### 3.3 Model performance comparisons

**Figure 3:**
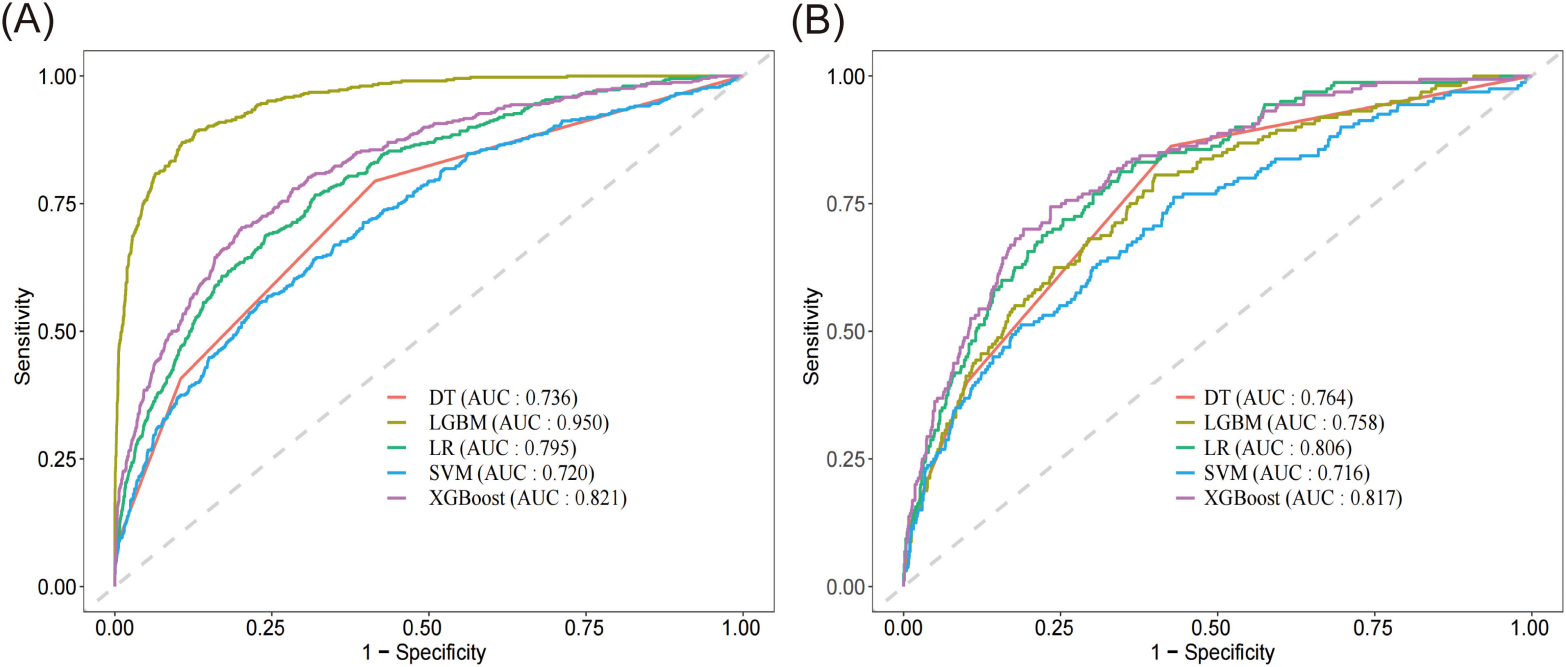
Receiver operating characteristic curve and of the five models. **(A)** ROC of the training set. **(B)** ROC of the validation set. DT, decision tree; LGBM, light gradient boosting machine; LR, logistic regression; SVM, support vector machine; XGBoost, extreme gradient boosting.

Based on the evaluation results presented in **Table 2** and **Figure 3**, we conducted a comprehensive analysis of the performance of five machine learning algorithms across various metrics on both the training and validation datasets. The LightGBM algorithm demonstrated superior performance on the training set, achieving an AUC of 0.950, accuracy of 0.882, sensitivity of 0.892, specificity of 0.871, recall of 0.892, and an F1 score of 0.644, surpassing other algorithms across all metrics. However, its performance on the validation set showed a notable decrease, particularly in AUC (0.758) and sensitivity (0.562), suggesting potential overfitting. On the other hand, the XGBoost algorithm showed relatively stable performance on both the training and validation sets, with AUC values of 0.821 (**Figure 3A**) and 0.817 (**Figure 3B**) respectively. The model demonstrated a strong capacity for generalization, as it attained the highest validation AUC while also striking a balanced compromise between accuracy (0.742), sensitivity (0.700), and specificity (0.784) on the validation set. The LR algorithm also showed stable performance across datasets, with a validation AUC of 0.806 and an accuracy of 0.727. The DT algorithm achieved a high accuracy (0.869) on the training set, but its performance decreased on the validation set, particularly in sensitivity, which dropped from 0.857 to 0.750. The SVM algorithm performed relatively weakly on both datasets, with AUC values of only 0.720 and 0.716. Considering the balance between model performance and generalizability, we proposed that the XGBoost algorithm was the most suitable candidate for further interpretability analysis.

**Table 2:** Evaluation of the performance of the five algorithm.

Algorithm	Data set	AUC	Accuracy	Sensitivity	Specificity	Recall	F1 score
LR	Train	0.795	0.724	0.767	0.681	0.767	0.389
	Validation	0.806	0.727	0.794	0.660	0.794	0.363
DT	Train	0.736	0.869	0.857	0.880	0.857	0.135
	Validation	0.764	0.820	0.750	0.891	0.750	0.136
XGBoost	Train	0.821	0.751	0.703	0.798	0.703	0.457
	Validation	0.817	0.742	0.700	0.784	0.700	0.420
SVM	Train	0.720	0.663	0.556	0.770	0.556	0.356
	Validation	0.716	0.647	0.537	0.756	0.537	0.318
LightGBM	Train	0.950	0.882	0.892	0.871	0.892	0.644
	Validation	0.758	0.683	0.562	0.804	0.562	0.370

### 3.4 Model interpretation

**Figure 4:**
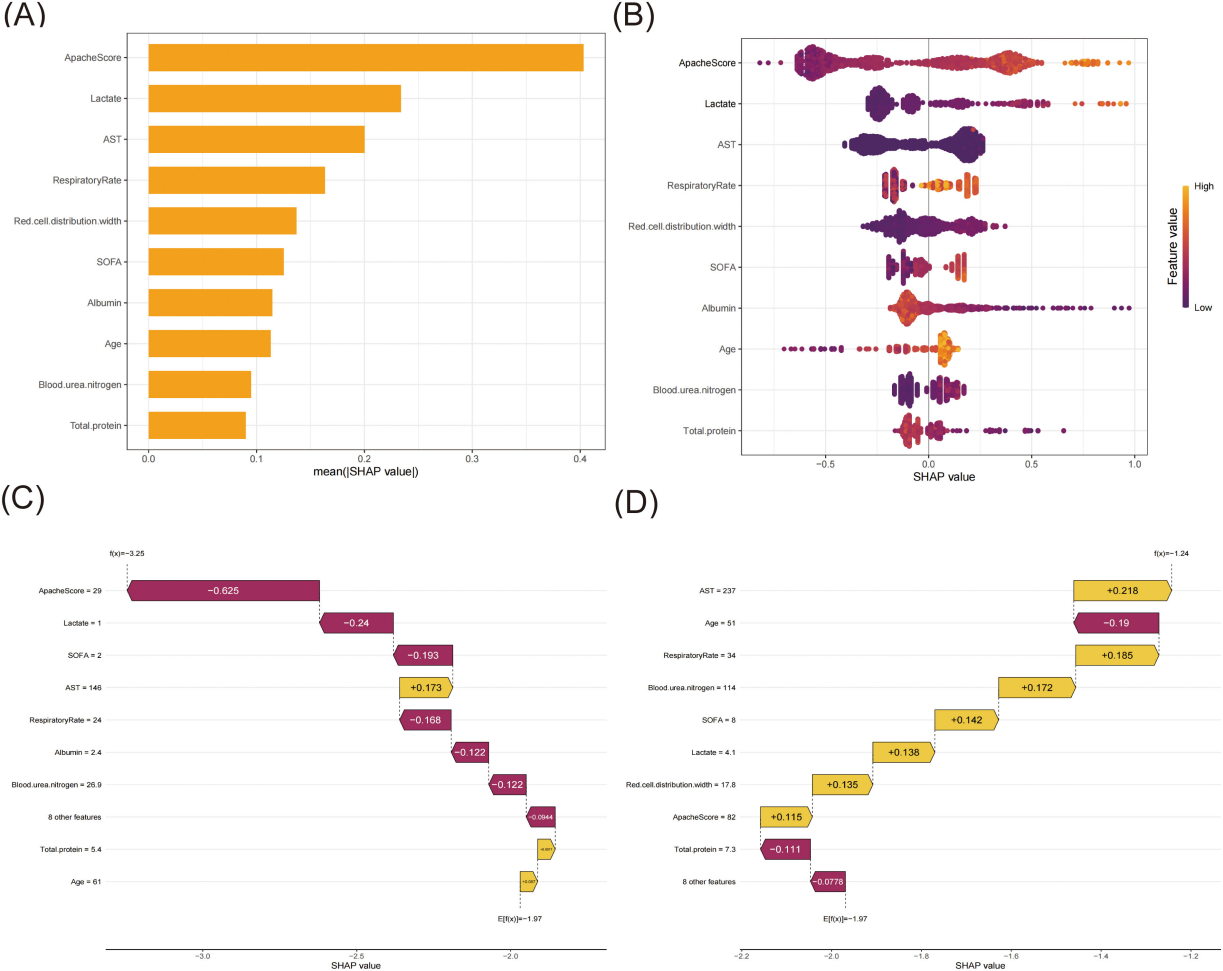
The SHAP analysis of the XGBoost model. **(A)** A bar plot displaying the mean SHAP value for the top ten variables. **(B)** The beeswarm plots displayed the distribution of the top ten variables, with variable values represented by different colors. Each sample was represented by a colored point. The x-axis represented the SHAP value, while the color coding indicated the feature values. **(C)** SHAP waterfall plot for case 1. **(D)** SHAP waterfall plot for case 2.

The XGBoost model was ultimately used to predict 28-day all-cause mortality in sepsis patients, along with conducting an analysis of model interpretability. Based on SHAP analysis (**Figures 4A, B**), the APACHE score demonstrated the highest predictive importance (mean SHAP value > 0.40), followed by the serum lactate level and AST (SHAP value ≥ 0.20). The respiratory rate showed a moderate influence (SHAP value > 0.15), with red cell distribution width and SOFA score exhibiting comparable impacts. Other clinical features, including albumin, age, blood urea nitrogen, and total protein, also significantly contributed. These variables demonstrated substantial contributions to the model’s predictive performance. Higher values of APACHE score, lactate level, and AST were associated with increased mortality risk, while elevated albumin and total protein levels were protective factors. The SHAP dependence plots (**Supplementary Figure S1**) revealed the relationship between feature values and their impact on model predictions. When the SHAP values turned positive, these variables were found to enhance the predicted outcomes.

To elucidate the model’s decision-making process at the individual level, we performed local interpretability analysis using SHAP waterfall plots on two randomly selected representative cases from the training set (**Figures 4C, D**). For one case (**Figure 4C**), the model predicted a markedly decreased mortality risk (final prediction f(x) = -3.25 compared to the base value E[f(x)] = -1.97) for this 61-year-old patient. The most substantial protective factor was the notably low APACHE score (29, SHAP value: -0.625), indicating relatively mild disease severity, followed by low serum lactate (1.0 mmol/L, SHAP value: -0.24) suggesting adequate tissue perfusion, and a low SOFA score (2, SHAP value: -0.193) reflecting minimal organ dysfunction. Notably, the respiratory rate remained stable (24 breaths/min, SHAP value: -0.168). Despite elevated AST levels (146 U/L, SHAP value: +0.173) indicating some degree of hepatic dysfunction, other parameters remained favorable. In contrast, Case 2 (**Figure 4D**) presented a 51-year-old patient, with the model suggesting an increased mortality risk (final prediction f(x) = -1.24 compared to the base value E[f(x)] = -1.97). The significant risk factors included notable hepatic dysfunction (AST: 237 U/L, SHAP value: +0.218), a high APACHE score (82, SHAP value: +0.115), and an increased red cell distribution width (17.8%, SHAP value: +0.135). The patient presented with significant organ dysfunction (SOFA score: 8, SHAP value: +0.142), elevated serum lactate (4.1 mmol/L, SHAP value: +0.138), and markedly elevated blood urea nitrogen (114 mg/dL, SHAP value: +0.172). The elevated respiratory rate (34 breaths/min, SHAP value: +0.185) suggested respiratory distress. Despite these risk factors, the total protein remained within the normal range (7.3 g/dL, SHAP value: -0.111). The SHAP values quantify each feature’s contribution, with positive values (yellow bars) indicating risk-increasing factors and negative values (magenta bars) representing protective effects.

## 4 Discussion

This study developed and validated a machine learning model to predict 28-day all-cause mortality in ICU patients with sepsis using data from the eICU Collaborative Research Database. Among the five algorithms tested (logistic regression, decision tree, extreme gradient boosting, support vector machine, and light gradient boosting machine), the XGBoost model demonstrated the most stable and balanced performance, with an AUC of 0.821 on the training set and 0.817 on the validation set. The model identified APACHE score, serum lactate levels, and AST as the top three predictors of mortality risk, followed by other important factors such as respiratory rate, red cell distribution width, SOFA score, albumin, age, blood urea nitrogen, and total protein. Through SHAP analysis, the study emphasized model interpretability, clarifying the specific contribution of each feature to the prediction results, thereby enhancing the model’s potential for clinical application in sepsis management.

By combining LASSO regression with the Boruta algorithm, we were able to greatly improve the reliability of risk factor identification. LASSO effectively reduced model complexity and mitigated overfitting (McNeish, 2015), while Boruta provided a comprehensive evaluation of feature importance, considering potential variable interactions (Maurya et al., 2023). This method enabled us to identify key predictors that are both statistically significant and clinically relevant.

The XGBoost algorithm showed optimal performance in analyzing complex eICU data, as the model identified the combination of predictors reflecting the multi-system nature of sepsis. The prominence of APACHE and SOFA scores as top predictors reaffirmed the value of these comprehensive scoring systems in assessing disease severity (Huang et al., 2022). While the APACHE-IV scoring system has demonstrated satisfactory discriminative capability in predicting 30-day mortality among patients with ischemic stroke or intracerebral hemorrhage (van Valburg et al., 2024), its performance in predicting intensive care unit length of stay among sepsis patients has been notably limited (Zangmo and Khwannimit, 2023). In addition, our study found that a higher level of lactate was a major risk factor for 28-day mortality in the ICU. Previous studies found that lactate levels, both at admission and after 24 hours, were valuable predictors of in-hospital mortality in sepsis patients (Baysan et al., 2022). Lactate played a dual role in inflammatory processes, acting as both a pro-inflammatory mediator by activating inflammatory pathways and cytokine release, and as an anti-inflammatory agent by modulating immune cell function and promoting tissue repair (Manosalva et al., 2021). Sepsis-associated microcirculatory dysfunction leads to tissue hypoperfusion and oxygen deficit, resulting in increased lactate production through anaerobic glycolysis and impaired oxygen utilization (Gattinoni et al., 2019). The inclusion of biochemical indicators such as AST, albumin, and total protein highlights the critical role of liver function, nutritional status, and overall metabolic state in sepsis prognosis. Furthermore, the identification of unusual predictors, such as red cell distribution width (RDW), demonstrated our model’s ability to detect subtle but important signs of inflammation in sepsis and predict 28-day mortality. A multicenter study found that RDW was associated with mortality in sepsis patients, proposed 16% as the optimal RDW cutoff for predicting in-hospital mortality (Dankl et al., 2022). In hospitalized patients over 60 years old, RDW was significantly associated with higher in-hospital mortality, increased 30-day readmission rates, and longer hospital stays (Kim et al., 2022). In sepsis, systemic inflammation marked by elevated cytokines like IL-6 and TNF-α impairs erythropoiesis, leading to increased RDW, which correlates with disease severity and poor outcomes (Pierce and Larson, 2005; Salvagno et al., 2015). Oxidative stress damaged red cell membranes and reduced erythrocyte lifespan when suffering from sepsis, leading to increased production of new red blood cells of varying sizes, which resulted in elevated RDW (Friedman et al., 2004; Salvagno et al., 2015).

The selection of performance metrics in our study was carefully considered to provide a comprehensive evaluation of the model’s clinical utility. Among all the algorithms, LightGBM demonstrated the strongest performance in the training set with an AUC of 0.950, though its performance in the validation set (AUC = 0.758) suggests some degree of overfitting. XGBoost showed the most consistent performance between the training and validation sets (AUC = 0.821 and 0.817, respectively), indicating robust generalizability. The XGBoost achieved a sensitivity of 0.700 in the validation set, meaning it correctly identified 70% of high-risk patients who may require immediate intensive intervention. Its specificity of 0.784 indicated good capability in identifying lower-risk patients.

To address the interpretability challenge of ML models, we employed SHAP values to provide transparent insights into the XGBoost model’s decision-making process. SHAP analysis revealed that APACHE score, lactate level, and AST were the top three predictors of 28-day all-cause mortality in ICU sepsis patients. The combination of elevated APACHE scores and high lactate levels showed a synergistic effect in predicting poor outcomes, while normal AST levels combined with low APACHE scores were associated with better survival probability. Furthermore, our case analysis validated these findings, demonstrating the model’s practical application in clinical settings.

This study had several strengths and limitations. Our use of advanced machine learning techniques, including XGBoost, allowed for the construction of complex models with powerful computational and fitting capabilities. The application of the SHAP analysis enhanced model interpretability, providing clinicians with insights into the decision-making process. The inclusion of 4,564 patients from multiple centers in the eICU database helped to increase the generalizability of our results. However, the study’s retrospective nature introduced potential biases. The lack of prospective validation in clinical trials limited our ability to determine the model’s exact real-world performance. Additionally, while we included a comprehensive set of variables, some potentially important factors, such as pre-ICU immobilization status, were not available in the database and thus not incorporated into our model. To address this limitation, we are currently carrying out a multicenter study that includes pre-ICU immobilization status among its assessment indicators. Additionally, our team is working on an intelligent prediction platform designed to help clinicians accurately predict the 28-day mortality risk for ICU sepsis patients. External validation through this ongoing research and future prospective studies will help confirm the model’s generalizability and clinical utility across diverse settings.

## 5 Conclusion

Machine learning models effectively predicted 28-day ICU mortality in sepsis patients. Among the five constructed models, the XGBoost model proved to be the most stable and effective, enabling early identification of high-risk sepsis mortality patients and facilitating individualized interventions to alleviate patient burden.

## Data Availability

The original contributions presented in the study are included in the article/**Supplementary Material**. Further inquiries can be directed to the corresponding authors.
