# Supplementary figures and images for: Interpretable machine learning-based prediction of 28-day mortality in ICU patients with sepsis: a multicenter retrospective study
Source: Front Cell Infect Microbiol. 2025 Jan 8;14:1500326. doi: 10.3389/fcimb.2024.1500326 (PMC11751000; doi:10.3389/fcimb.2024.1500326)

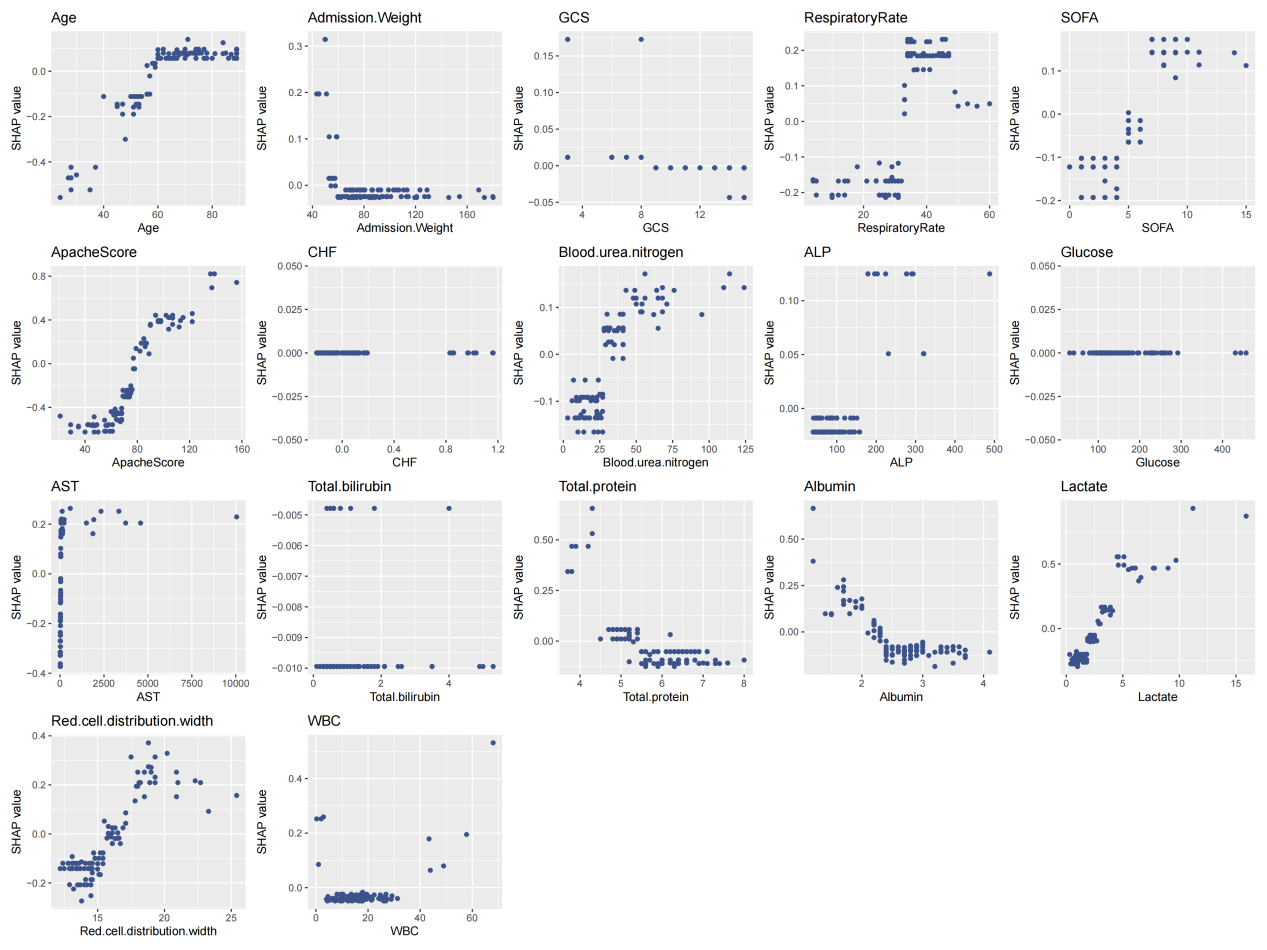


**Figure S1** SHAP feature dependence plots based on XGBoost Model.

Supplement: Supplementary file 2 [file DataSheet1.docx]
